# Natural History of Malignant Bone Disease in Hepatocellular Carcinoma: Final Results of a Multicenter Bone Metastasis Survey

**DOI:** 10.1371/journal.pone.0105268

**Published:** 2014-08-29

**Authors:** Daniele Santini, Francesco Pantano, Ferdinando Riccardi, Giovan Giuseppe Di Costanzo, Raffaele Addeo, Francesco Maria Guida, Mariella Spalato Ceruso, Sandro Barni, Paola Bertocchi, Sara Marinelli, Paolo Marchetti, Antonio Russo, Mario Scartozzi, Luca Faloppi, Matteo Santoni, Stefano Cascinu, Evaristo Maiello, Franco Silvestris, Marco Tucci, Toni Ibrahim, Gianluca Masi, Antonio Gnoni, Alessandro Comandone, Nicola Fazio, Alessandro Conti, Ilaria Imarisio, Salvatore Pisconti, Elisa Giommoni, Saverio Cinieri, Vincenzo Catalano, Vincenzo Ostilio Palmieri, Giovanni Infante, Michele Aieta, Antonio Trogu, Cosmo Damiano Gadaleta, Anna Elisabetta Brunetti, Vito Lorusso, Nicola Silvestris

**Affiliations:** 1 Medical Oncology Unit - University Campus Bio-Medico, Rome, Italy; 2 Medical Oncology Unit, Cardarelli Hospital, Naples, Italy; 3 Liver Unit, Cardarelli Hospital, Naples, Italy; 4 Medical Oncology Unit, ‘San Giovanni di Dio’ Frattamaggiore Hospital, Frattammaggiore, Italy; 5 Medical Oncology Unit, Treviglio-Caravaggio Hospital, Treviglio, Italy; 6 Medical Oncology Unit, Fondazione Poliambulanza, Brescia, Italy; 7 Department of Medical and Surgical Sciences, S.Orsola-Malpighi Hospital, University of Bologna, Bologna, Italy; 8 Department of Medical Oncology, University of Rome La Sapienza, Santa Andrea Hospital, Rome, Italy; 9 Section of Medical Oncology, Department of Surgical, Oncological and Stomatological Disciplines, University of Palermo, Palermo, Italy; 10 Clinica di Oncologia Medica, AOU Ospedali Riuniti-Università Politecnica delle Marche, Ancona, Italy; 11 Medical Oncology Unit – Hospital “Casa Sollievo della Sofferenza”, San Giovanni Rotondo, Italy; 12 Department of Biomedical Sciences and Human Oncology, University of Bari Aldo Moro, Bari, Italy; 13 Istituto Scientifico Romagnolo per lo Studio e la Cura dei Tumori (IRST), IRCCS- Osteoncology and Rare Tumors Center, Meldola, Italy; 14 Division of Medical Oncology 2, Azienda Ospedaliero-Universitaria Pisana, Pisa, Italy; 15 Medical Oncology Unit – Hospital of Lecce, Lecce, Italy; 16 Department of Oncology, Gradenigo Hospital and Gruppo Piemontese Sarcomi, Turin, Italy; 17 Unit of Gastrointestinal and Neuroendocrine Tumor, European Institute of Oncology, Milan, Italy; 18 Department of Clinical and Specialist Sciences, Urology, Polytechnic University of the Marche Region, AOU Ospedali Riuniti Umberto I-GM Lancisi and G Salesi, Ancona, Italy; 19 Medical Oncology, Fondazione IRCCS Policlinico S. Matteo, Pavia, Italy; 20 Medical Oncology Unit – S.G. Moscati Hospital ASL TA/1, Taranto, Italy; 21 Medical Oncology Unit – Hospital Careggi, Florence, Italy; 22 Medical Oncology Department & Breast Unit - Hospital of Brindisi and Medical Oncology Department - European Institute of Oncology, Milan, Italy; 23 Medical Oncology, A.O. “Ospedali Riuniti Marche Nord”, Presidio S. Salvatore, Pesaro, Italy; 24 Department of Biomedical Sciences and Human Oncology, Clinica Medica “A. Murri”, University of Bari, Bari, Italy; 25 U.O. Infectious Disease, P.O. Bisceglie, Bisceglie, Italy; 26 Centro di Riferimento Oncologico della Basilicata IRCCS, Rionero in Vulture, Italy; 27 Medical Oncology unit – Hospital of Aosta, Aosta, Italy; 28 Interventional Radiology Unit with Integrated Section of Translational Medical Oncology – National Cancer Institute “Giovanni Paolo II”, Bari, Italy; 29 Medical Oncology Unit – National Cancer Institute “Giovanni Paolo II”, Bari, Italy; IRCCS Istituto Oncologico Giovanni Paolo II, Italy

## Abstract

**Background:**

Bone is an uncommon site of metastasis in patients with advanced hepatocellular carcinoma (HCC). Therefore, there are few studies concerning the natural history of bone metastasis in patients with HCC.

**Patients and Methods:**

Data on clinicopathology, survival, skeletal-related events (SREs), and bone-directed therapies for 211 deceased HCC patients with evidence of bone metastasis were statistically analyzed.

**Results:**

The median age was 70 years; 172 patients were male (81.5%). The median overall survival was 19 months. The median time to the onset of bone metastasis was 13 months (22.2% at HCC diagnosis); 64.9% patients had multiple bone metastases. Spine was the most common site of bone metastasis (59.7%). Most of these lesions were osteolytic (82.4%); 88.5% of them were treated with zoledronic acid. At multivariate analysis, only the Child Score was significantly correlated with a shorter time to diagnosis of bone metastases (*p* = 0.001, HR = 1.819). The median survival from bone metastasis was 7 months. At multivariate analysis, HCC etiology (*p* = 0.005), ECOG performance status (*p* = 0.002) and treatment with bisphosphonate (*p* = 0.024) were associated with shorter survival after bone disease occurrence. The site of bone metastasis but not the number of bone lesions was associated with the survival from first skeletal related event (SRE) (*p* = 0.021) and OS (*p* = 0.001).

**Conclusions:**

This study provides a significant improvement in the understanding the natural history of skeletal disease in HCC patients. An early and appropriate management of these patients is dramatically needed in order to avoid subsequent worsening of their quality of life.

## Introduction

Hepatocellular carcinoma (HCC) is the sixth most prevalent cancer worldwide and the third leading cause of cancer-related death, although its geographical distribution is heterogeneous with the highest incidence in sub-Saharan Africa and Eastern Asia [1]. The choice of its therapy is related to the stage of the disease, severity of the underlying liver disease, and clinical expertise. Unfortunately, two thirds of patients are diagnosed at an advanced stage, when prognosis is poor with 5-year survival rates of less than 20% [2].

HCC is less likely to develop distant metastases, even in the inoperable stage, compared to other solid tumors with the lung as the most common site of localization [3]. Although bone involvement is reported as uncommon in HCC, its incidence has significantly increased in the last decade due to the improvement of overall survival of these patients [4], [5]. One recent study considering 342 HCC patients reported skeletal invasion in approximately 25% of extrahepatic metastases [6].

Axial skeleton is the most frequent localization of bone metastases with a prognostic correlation of the time between the primary HCC occurrence and bone metastases detection [7]. They are mainly osteolytic resulting in significant morbidity and reduced quality of life for patients from the associated skeletal-related events (SRE; defined as pathological fracture, the need for radiotherapy or surgery to bone, spinal cord compression, and hypercalcemia) [8]. Radiotherapy is the most common SRE, playing a role in bone pain palliation, mostly for patients whose liver failure can be associated with a reduced patients’ opioid tolerance. In most of cases, higher radiotherapy doses are required due to the presence of soft tissue masses in addition to bone involvement [9]. Indeed, few retrospective studies evaluated the use of biphosphonates in HCC-bone metastases [4]. Low doses of sorafenib have been associated to long term progression free survival in some patients [10].

Finally, herein we report the results of the largest multicenter study investigating the natural history (and their clinical management) of bone metastases from HCC.

## Patients and Methods

### Ethics Statement

This multicentre retrospective observational study has been approved by the Ethics Committee of the coordinator centre (National Cancer Institute of Bari). According to our Ethics Committee, a written consent was not needed. In fact, this is a retrospective observational study considering only died patients whose recruitment in the survey did not influenced their treatment.

### Study design

This retrospective, observational multicentre study aimed at defining the natural history of HCC patients with bone metastasis was conducted in 23 Italian hospital centres in which these patients received diagnosis and treatment of disease from January 1993 to May 2013. Data were collected from HCC patients of all ages who received standard treatments in accordance with each own treating physician’s practice and were not included neither in clinical trials nor experimental protocols. Moreover, patients had at least one bone metastasis during the course of their disease and died of HCC or HCC-related complications. In details, patients were identified as having bone metastasis if two of the following criteria were satisfied: physician reported bone metastasis; bone metastasis identified by bone scan; record of radiotherapy to bone as a palliative therapy; identification of bone metastasis by other imaging assessment (e.g. standard x-rays, computed tomography scans, or magnetic resonance imaging of the skeleton). Data were collected throughout the disease course and during all cancer treatments, including surgery, radiation therapy, locaregional therapies, chemotherapy, and biological therapies. Variables assessed included age, sex, aetiology, grading, Child score at diagnosis, presence and type of locoregional treatment, the median value of Alpha-fetoprotein (AFP) at diagnosis, number and sites of bone metastasis, visceral metastases, ECOG performance status (PS) at the moment of bone metastases diagnosis, time to appearance of bone metastasis, times to first and subsequent SREs (from diagnosis of bone metastasis), SRE types, survival after bone metastases diagnosis and after first SRE, systemic therapy with Sorafenib and type and time of bisphosphonate therapy.

### Statistical analysis

Descriptive statistics were used for patient demographics and incidence of SREs. All survival intervals were determined by the Kaplan-Meier method. The differences in survival according to clinical parameters or treatment were evaluated by the log-rank test and described by the Kaplan–Meier method unless otherwise specified. In the univariate model, all the clinical variables were evaluated as predictors for shorter time to bone metastasis, shorter time from bone metastases to SRE and shorter time from bone metastases to death. Patients who did not have a recorded date for a specific event were censored at the date of death. Finally, the Cox proportional hazards model was applied to the multivariate survival analysis. All the significant variables in the univariate model were used to build the multivariate model of survival, and median values were derived from whole-month values rather than fractions. SPSS software (version 20.00; SPSS, Chicago, IL) was used for statistical analysis. A *p* value<0.05 was considered statistically significant.

## Results

### Patient characteristics

We retrospectively enrolled 211 patients died from HCC with bone metastasis. Of them, 172 patients were male (81.5%). The median age was 70 years (SD +/−9). Tumor etiology of HCC was HBV related in 35/211 (16.5%) patients, HCV related in 110/211 (52.1%) patients, alcohol related in 20/211 (9.4%) patients and other causes-related in 46/211 (21.8%) patients. The subgroup with Child A was the largest (69.8%) patients, followed by the subgroup with Child B (22.0%) and Child C (8.2%) patients. The majority of patients (132/211; 62.6%) was treated with Sorafenib. The remaining baseline characteristics as grading, presence and type of locoregional treatment, presence of visceral metastases and the median value of AFP at diagnosis were summarized in Table 1.

**Table 1:** Baseline characteristics.

Baseline characteristics (Total N = 211)	Frequency (pts/total applicable)	P (%)
**Age**		
<70 Years	111/205	54.1
>70 Years	94/205	45.9
**Gender**		
Male	172/211	81.5
Female	39/211	18.5
**Aetiology**		
HBV-related	35/211	16.5
HCV-related	110/211	52.1
Alcohol-related **cirrosi**	20/211	9.4
Other	46/211	21.8
**Grading**		
G1	28/98	28.6
G2	30/98	30.6
G3	40/98	40.8
**Locoregional Treatment**		
No	149/211	70.6
Yes	62/211	29.4
**Type of locoregional Treatment**		
Surgery	50/149	33.5
Interventional Radiology	89/149	66.5
**Type of interventional Radiology**		
RFA	23/89	25.8
TACE	57/89	64.0
PEI	9/89	10.1
**CHILD Score**		
A	133/191	69.8
B	42/191	22.0
C	16/191	8.4
**Visceral Metastasis**		
Yes	151/211	71.6
No	60/211	28.4
**AFP (cut-off value of 200 ng/mL at diagnosis)**		
<200 ng/ml	97/149	65.1
>200 ng/ml	52/149	34.9
**AFP (median value at diagnosis)**		
<43 ng\ml	75/149	50.4
>43 ng/ml	74/149	49.6
**Sorafenib Treatment**		
Yes	132/211	62.6
No	79/211	37.4

*Abbreviations: n, number; pts, patients; HBV, Hepatitis B virus; HCV, Hepatitis C virus.*

### Skeletal metastases

The ECOG PS at the moment of bone metastasis diagnosis was 0 for 51/194 patients (26.3%), 1 for 78/194 (40.2%), 2 for 50/194 (25.8%), 3 for 15/194 (7.7%) and unknown in 17 patients (8%). One hundred and sixty one patients (77.8%) developed bone metastasis after HCC diagnosis while 46 patients (22.2%) showed bone metastasis at the time of HCC diagnosis; 137 of 211 patients (64.9%) had multiple bone metastases and the remaining 74/211 patients (35.1%) showed single lesion. Spine were the most common site of bone metastasis (126/211; 59.7%) followed by hip (74/211; 35.1%) and long bones (41/211; 19.4%) and are consistent with previous reports. Osteolytic lesions (169/205; 82.4%) were far more prevalent in this group than the mixed ones (20/205; 9.8%) and osteoblastic lesions (16/205; 7.8%). More than half of the patients (127/211; 60.1%) experienced at least one SRE while, two and three SREs have been reported in 18.9% (40/211) and 2.8% (6/211) of patients, respectively (Table 2). Considering only the first SRE, radiotherapy to bone is the most common (70/127 patients; 55.1%), followed by pathologic fracture (31/127; 24.4%), spinal cord compression (12/127; 9.4%), surgery to bone (7/127; 5.5%) and hypercalcemia (7/127; 5.5%) while for second and third SRE, radiotherapy to bone also had the greater incidence with 47.5% (19/40) and 66.6% (4/6) respectively. Equally, considering all the different SREs, radiotherapy to bone is the most common SRE (53.7% of all events), followed by pathologic fracture (20.8%), spinal cord compression (9.8%), surgery to bone (8%) and hypercalcemia (7.5%). Among the 211 patients with bone metastasis, 105/211 (49.7%) patients received therapy with bisphosphonate: 93 patients (88.5%) were treated with Zoledronic Acid, 6 patients (5.6%) with Pamidronate and 7 patients (6.6%) with other agents, respectively. No patient developed osteonecrosis of the jaw (ONJ) (Table 2).

**Table 2:** Skeletal metastases.

Skeletal metastases (total n = 211)	Frequency (pts)	Percentage (%)
**ECOG PS (at time of Bone Metastasis)**		
**0**	51/194	26.3
**1**	78/194	40.2
**2**	50/194	25.8
**3**	15/194	7.7
**Bone Metastasis at diagnosis**		
Yes (synchronous)	161/207	77.8
No (metachronous)	46/207	22.2
**Bone Lesion Type**		
Osteolytic	169/205	82.4
Osteoblastic	16/205	7.8
Mixed	20/205	9.8
**Number of Bone Metastasis**		
1	74/211	35.1
>1	137/211	64.9
**Bone Metastasis Localization**		
Spine	126/211	59.7
Long Bones	41/211	19.4
Hip	74/211	35.1
Other Sites	61/211	28.9
**Total SRE Number**		
0	84/211	39.9
1	127/211	60.1
2	40/211	18.9
3	6/211	2.8
**First SRE Type**		
Pathological Fracture	31/127	24.4
Hypercalcemia	7/127	5.5
Spinal Cord Compression	12/127	9.4
Surgery to Bone	7\127	5.5
Radiation to Bone	70/127	55.1
**Second SRE Type**		
Pathological Fracture	5/40	12.5
Hypercalcemia	6/40	15
Spinal Cord Compression	5/40	12.5
Surgery to Bone	5/40	12.5
Radiation to Bone	19/40	47.5
**Third SRE Type**		
Pathological Fracture	0/6	0.0
Hypercalcemia	0/6	0.0
Spinal Cord Compression	0/6	0.0
Surgery to Bone	2/6	33.4
Radiation to Bone	4/6	66.6
**Biphosphonate Treatment**	105/211	49.7
Zoledronic Acid	93/105	88.5
Pamidronate	6/105	5.6
Other	7/105	6.6

### Predictive factors of onset of bone metastasis

The median time to the onset of bone metastasis was 13 months (CI 95% 9.29–16.71 months). At univariate analysis (Table 3), the median time to the onset of skeletal disease was significantly shorter according to type of locoregional treatment (17 months for interventional radiology vs. 10 months for surgery; CI 95% 8.41−11.58 and 10.82−23.17, respectively; *p = *0.037), Child Score (*p*<0.001) and in patients with higher median AFP at diagnosis (*p = *0.040). At multivariate analysis, only the Child Score was confirmed and independently correlated with a shorter time to diagnosis of bone metastases (Table 3) (*p = *0.001; HR: 1.819).

**Table 3:** Predictive factors of onset of bone metastasis.

VARIABLES	MEDIAN TIME(MONTHS)	p VALUE(uni variate)	p VALUE(multivariate)	HAZARDRATIO (HR)
**Age**		0.604		
<70 Years	12.0 (9.37–14.63)			
>70 Years	16.0 (11.11–20.89)			
**Gender**		0.807		
Male	15.0 (11.77–18.24)			
Female	12.0 (9.13–14.87)			
**Aetiology**		0.715		
HBV-related	12.0 (3.84–20.16)			
HCV-related	14.0 (10.35–17.65)			
Alcohol-related cirrhosis	15.0 (9.91–20.09)			
Other	8.0 (5.37–10.63)			
**Grading**		0.254		
G1	24.0 (12.04–35.96)			
G2	19.0 (12.11.25.90)			
G3	10.0 (2.70–17.30)			
**Locoregional Treatment**		0.360		
No	14.0 (6.43–21.57)			
Yes	14.0 (10.96–17.04)			
**Type of locoregional Treatment**		***0.037***	0.988	0.994
Surgery	10.0 (8.41–11.59)			
Interventional Radiology	17.0 (10.83–23.17)			
**Type of interventional Radiology**		0.716		
RFA	12.0 (2.61–21.39)			
TACE	16.0 (10.35–21.65)			
PEI	24.0 (11.53–36.47)			
**CHILD Score (at time of Bone Metastasis)**		***0.000***	***0.001***	***1.819***
A	16.0 (10.40–21.60)			
B	12.0 (9.32–14.68)			
C	7.0 (4.93–9.07)			
**AFP (at diagnosis)**		***0.040***	0.157	1.346
<43 ng\ml	17.0 (12.40–21.61)			
>43 ng/ml	12.0 (8.47–15.53)			
**Bone Lesion Type**		0.932		
Osteolytic	14.0 (11.27–16.73)			
Ostroblastic	16.0 (0.00–37.69)			
Mixed	14.0 (3.82–24.18)			

*Abbreviations: CI, Confidence Interval; AFP, Alpha-fetoprotein; RFA, radiofrequency ablation; TACE, Transcatheter arterial chemoembolization; PEI, percutaneous ethanol injection.*

### Predictive factors of survival after bone metastases diagnosis

The median survival from the diagnosis of bone metastasis was 7 months (CI 95% 5.36–8.64 months). The univariate analysis, reported in Table 4, demonstrates that the median survival after diagnosis of bone metastases was significantly shorter according to HCC etiology (4 months for HBV, 7 months for HCV, 9 months for alcohol related and 8 months for other causes; CI 95% 2.18−5.81, 5.61−8.38, 4.73−13.26 and 5.10−10.89 months, respectively; *p* = 0.003), ECOG PS (*p* = 0.002), in patients with bone metastasis localized to spine (*p* = 0.006) and did not receive any bisphosphonate treatment (*p* = 0.001). Notably, at multivariate analysis (Table 4) all these parameters were confirmed and independently correlated with a shorter survival after bone disease occurrence (*p* = 0.005 with HR: 0.785 for etiology; *p* = 0.002 with HR: 1.341 for ECOG PS and *p* = 0.024 with HR: 0.669 for bisphosphonate treatment, respectively), excluding bone metastasis to spine (*p* = 0.075 with HR: 1.339).

**Table 4:** Predictive factors of survival after bone metastases diagnosis.

VARIABLES	MEDIAN TIME(MONTHS)	p VALUE(uni variate)	p VALUE(multivariate)	HAZARDRATIO (HR)
**Age**		0.349		
<70 Years	7.0 (5.83–8.18)			
>70 Years	6.0 (4.35–7.65)			
**Gender**		0.612		
Male	6.0 (5.07–6.93)			
Female	9.0 (7.33–10.67)			
**Aetiology**		***0.003***	***0.005***	***0.785***
HBV-related	4.0 (2.18–5.82)			
HCV-related	7.0 (5.61–8.39)			
Alcohol-related cirrhosis	9.0 (4.73–13.27)			
Other	8.0 (5.10–10.90)			
**Grading**		0.866		
G1	5.0 (3.38–6.62)			
G2	6.0 (3.74–8.26)			
G3	6.0 (4.04–7.96)			
**Locoregional Treatment**		0.104		
No	6.0 (4.93–7.07)			
Yes	7.0 (5.97–8.03)			
**Type of locoregional Treatment**		0.644		
Surgery	8.0 (6.23–9.77)			
Interventional Radiology	7.0 (5.72–8.28)			
**Type of interventional Radiology**		0.366		
RFA	7.0 (4.76–9.24)			
TACE	7.0 (5.25–8.76)			
PEI	7.0 (0.00–19.47)			
**CHILD Score** **(at time of Bone Metastasis)**		0.245		
A	6.0 (4.95–7.06)			
B	6.0 (3.93–8.07)			
C	9.0 (5.08–12.92)			
**AFP (at diagnosis)**		0.549		
<43 ng\ml	7.0 (5.22–8.78)			
>43 ng/ml	7.0 (5.93–8.07)			
**Visceral Metastasis**		0.615		
Yes	7.0 (4.14–9.86)			
No	7.0 (6.03–7.97)			
**ECOG PS** **(at time of Bone Metastasis)**		***0.002***	***0.002***	***1.341***
0	8.0 (5.28–10.72)			
1	8.0 (6.75–9.25)			
2	6.0 (4.85–7.15)			
3	4.0 (0.33–7.67)			
**Bone Lesion Type**		0.608		
Osteolytic	7.0 (6.05–7.95)			
Ostroblastic	9.0 (7.70–10.30)			
Mixed	7.0 (4.16–9.84)			
**Bone metastasis - Spine**		***0.006***	0.075	1.339
No	9.0 (7.34–10.66)			
Yes	6.0 (5.05–6.95)			
**Bone Metastasis - Long Bones**		0.806		
No	7.0 (6.11–7.89)			
Yes	6.0 (2.48–9.52)			
**Bone Metastasis - Hip**		0.428		
No	7.0 (5.95–8.05)			
Yes	6.0 (3.65–8.35)			
**Bone Metastasis - Other**		0.941		
No	7.0 (5.86–8.14)			
Yes	7.0 (5.60–8.40)			
**First SRE Type**		0.268		
Pathologica Fracture	7.0 (3.90–10.10)			
Hypercalcemia	9.0 (7.83–10–17)			
Spinal Cord Compression	4.0 (0.00–9.09)			
Surgery to Bone	9.0 (7.83–10.17)			
Radiation to Bone	8.0 (6.55–9.45)			
**Biphosphonate Treatment**		***0.001***	***0.024***	***0.699***
No	5.0 (3.58–6.42)			
Yes	8.0 (6.78–9.23)			

### Predictive factors of survival after HCC diagnosis

Considering all patients included in this study (N = 211) the median overall survival time from diagnosis of HCC was 19 months (CI 95%, 15.62–22.38) while the median survival from the start of Sorafenib was 9 months (CI 95%, 7.44–10.56 months) and median time to progression was 5 months (CI 95%: 3.70–6.30 months). The univariate analysis, reported in Table 5, demonstrates that the median overall survival was significantly correlated to Grading (*p* = 0.019), Child score at diagnosis (*p* = 0.001), presence of bone metastasis localized to spine (*p* = 0.018) and absence of any locoregional treatment (*p*<0.001). At multivariate analysis, absence of locoregional treatment (*p*<0.001; HR: 0.265), Child score at diagnosis (*p* = 0.049; HR: 1.572) and presence of bone metastasis to spine (*p* = 0.001; HR = 2.281) were confirmed and independently correlated with a shorter overall survival (Table 5).

**Table 5:** Predictive factors of survival after HCC diagnosis.

VARIABLES	MEDIAN TIME[MONTHS (95% C.I.)]	p VALUE(univariate)	p VALUE(multivariate)	HAZARDRATIO (HR)
**Age**		0.818		
<70 Years	18.0 (14.45–21.55)			
>70 Years	20.0 (16.77–23.23)			
**Gender**		0.589		
Male	19.0 (14.06–23.94)			
Female	19.0 (15.48–22.52)			
**Aetiology**		0.077		
HBV-related	15.0 (11.18–18.82)			
HCV-related	24.0 (20.49–27.51)			
Alcohol-related cirrhosis	16.0 (4.27–27.73)			
Other	13.0 (9.00–17.01)			
**Grading**		***0.019***	0.137	1.250
G1	27.0 (12.31–41.69)			
G2	26.0 (22.50–29.50)			
G3	12.0 (9.52–14.48)			
**Locoregional Treatment**		***0.000***	***0.000***	***0.265***
No	8.0 (4.43–11.57)			
Yes	24.0 (22.59–25.41)			
**Type of locoregional Treatment**		0.594		
Surgery	21.0 (16.36–25.64)			
Interventional Radiology	24.0 (21.94–26.06)			
**Type of interventional Radiology**		0.180		
RFA	26.0 (17.03–34.97)			
TACE	24.0 (20.62–27.38)			
PEI	29.0 (12.37–45.63)			
**CHILD Score** **(at time of Bone Metastasis)**		***0.001***	***0.049***	***1.572***
A	21.0 (15.75–26.25)			
B	18.0 (14.91–21.10)			
C	16.0 (14.06–17.95)			
**AFP (at diagnosis)**		0.419		
<43 ng\ml	18.0 (12.03–23.97)			
>43 ng/ml	19.0 (14.36–23.64)			
**Visceral Metastasis**		0.091		
Yes	24.0 (19.85–28.15)			
No	18.0 (15.29–20.71)			
**Bone metastasis - Spine**		***0.018***	***0.001***	***2.281***
No	21.0 (16.87–25.13)			
Yes	19.0 (15.26–22.74)			
**Bone Metastasis - Long Bones**		0.639		
No	19.0 (15.65–22.35)			
Yes	21.0 (9.59–32.41)			
**Bone Metastasis - Hip**		0.840		
No	23.0 (20.29–25.71)			
Yes	15.0 (11.87–18.13)			
**Bone Metastasis - Other**		0.718		
No	19.0 (15.21–22.79)			
Yes	19.0 (11.00–27.00)			
**First SRE Type**		0.742		
Pathologica Fracture	21.0 (16.68–25.32)			
Hypercalcemia	19.0 (11.3–26.70)			
Spinal Cord Compression	24.0 (9.97–38.03)			
Surgery to Bone	18.0 (0.00–38.53)			
Radiation to Bone	18.0 (12.86–23.14)			
**Biphosphonate Treatment**		0.529		
No	18.0 (12.64–23.36)			
Yes	19.0 (16.35–21.65)			

### Skeletal outcomes and SREs in the overall population

The median number of SREs experienced by patients was one (range 0–3). Median survival after development of the first SRE was 8 months (CI 95% 7.17–8.20 months). The univariate analysis, reported in Table 6, demonstrates that the median survival after development of the first SRE was significantly shorter according to the type of first SRE (2 months for spinal cord compression, 3 months for surgery to bone, 4 months for pathological fracture, 5 months for hypercalcemia and for radiation to bone; CI 95%: 0.32–3.67, 0.00–7.80, 2.24–5.75, 0.00–10.13 and 3.79–6.20 months respectively; *p* = 0.024) and in patients that did not receive any locoregional treatment (*p*<0.001), bisphosphonate therapy (*p* = 0.001) and with presence of bone metastasis localized to spine (*p* = 0.027). At multivariate analysis, locoregional treatment and presence of bone metastastasis localizated to spine were confirmed and independently correlated with a shorter survival after development of the first SRE (*p* = 0.030; HR: 4.709 and *p*<0.001; HR: 12.280, respectively) (Table 6). The median time to first SRE after confirmed diagnosis of bone metastasis was 3 months (CI 95%, 2.27–3.73 months), indicative of the severity of bone metastasis in HCC. The median time to second SRE was 6 months and to third SRE was 9 months. At univariate analysis (Table 7), the median time to first SRE after confirmed diagnosis of bone metastasis was significantly shorter according to Child Score (*p*<0.001), ECOG PS (*p = *0.014) and in patients with presence of bone metastasis localized to Spine (*p* = 0.021) and in other site excluding hip, long bones and the same spine (*p* = 0.021). At multivariate analysis, only bone metastasis localized in other site which are not spine, hip and long bones were confirmed and independently correlated with a shorter time to first SRE after confirmed diagnosis of bone metastasis (*p* = 0.025; HR: 0.570). All data described are reported in Table 7.

**Table 6:** Predictive factors of survival after the development of the first SRE.

VARIABLES	MEDIAN TIME[MONTHS- (95% C.I.)]	p VALUE(univariate)	p VALUE(multivariate)	HAZARDRATIO (HR)
**Age**		0.872		
<70 Years	2.0 (0.63–3.37)			
>70 Years	2.0 (0.34–3.66)			
**SEX**		0.799		
Male	1.0 (0.00–2.21)			
Female	3.0 (1.67–4.32)			
**Aetiology**		0.339		
HBV-related	0.0 (0.00–0.00)			
HCV-related	3.0 (1.51− (4.49)			
Alcohol-related cirrhosis	2.0 (0.93–3.07)			
Other	0.0 (0.00–0.00)			
**Grading**		0.847		
G1	3.0 (0.06–5.94)			
G2	2.0 (0.00–5.96)			
G3	1.0 (0.00–2.16)			
**Locoregional Treatment**		***0.000***	***0.030***	***0.575***
No	0.0 (0.00–0.00)			
Yes	3.0 (1.91–4.10)			
**Type of locoregional Treatment**		0.330		
Surgery	4.0 (2.90–5.10)			
Interventional Radiology	2.0 (0.51–3.50)			
**Type of interventional Radiology**		0.089		
RFA	3.0 (1.21–4.79)			
TACE	2.0 (2.32–5.80)			
PEI	1.0 (0.00–10.70)			
**CHILD Score** **(at time of Bone Metastasis)**		0.509		
A	1.0 (0.00–3.49)			
B	3.0 (2.26–3.74)			
C	4.0 (2.06–5.95)			
**AFP (median value at diagnosis)**		0.186		
<43 ng\ml	3.0 (1.80–4.20)			
>43 ng/ml	1.0 (0.70–3.10)			
**Visceral Metastasis**		0.119		
Yes	1.0 (0.00–1.94)			
No	2.0 (1.06–2.95)			
**ECOG PS** **(at time of Bone Metastasis)**		0.115		
**0**	1.0 (0.00–3.34)			
**1**	3.0 (1.65–4.35)			
**2**	2.0 (0.00–4.31)			
**3**	0.0 (0.00–0.00)			
**Bone Lesion Type**		0.362		
Osteolytic	2.0 (0.44–3.57)			
Ostroblastic	2.0 (0.88–3.12)			
Mixed	2.0 (0.00–4.11)			
**Bone metastasis - Spine**		***0.027***	***0.000***	***2.049***
No	3.0 (1.30–4.70)			
Yes	1.0 (0.00–2.00)			
**Bone Metastasis - Long Bones**		0.191		
No	1.0 (0.00–2.16)			
Yes	4.0 (2.73–5.27)			
**Bone Metastasis - Hip**		0.382		
No	2.0 (0.84–3.16)			
Yes	2.0 (0.00–4.19)			
**Bone Metastasis - Other**		0.154		
No	3.0 (2.19–3.81)			
Yes	0.0 (0.00–0.00)			
**First SRE Type**		***0.024***	0.239	0.935
Pathologica Fracture	4.0 (2.24–5.76)			
Hypercalcemia	5.0 (0.00–10.13)			
Spinal Cord Compression	2.0 (0.33–3.67)			
Surgery to Bone	3.0 (0.00–7.80)			
Radiation to Bone	5.0 (3.80–6.20)			
**Biphosphonate Treatment**		***0.001***	0.727	0.931
No	0.0 (0.00–0.00)			
Yes	4.0 (2.99–5.02)			

**Table 7:** Predictive factors of onset of first SRE.

VARIABLES	MEDIAN TIME[MONTHS (95% C.I.)]	p VALUE(univariate)	p VALUE(multivariate)	HAZARDRATIO (HR)
**Age**		0.670		
<70 Years	8.0 (7.11–8.89)			
>70 Years	7.0 (5.33–8.67)			
**Gender**		0.403		
Male	8.0 (6.70–9.30)			
Female	8.0 (6.91–9.09)			
**Aetiology**		0.095		
HBV-related	8.0 (5.72–10.28)			
HCV-related	8.0 (7.11–8.89)			
Alcohol-related cirrhosis	7.0 (4.46–9.54)			
Other	11.0 (6.23–15.77)			
**Grading**		0.907		
G1	7.0 (4.29–9.71)			
G2	11.0 (4.78–17.22)			
G3	9.0 (6.85–11.15)			
**Locoregional Treatment**		0.239		
No	10.0 (5.61–14.39)			
Yes	7.0 (6.27–7.73)			
**Type of locoregional Treatment**		0.717		
Surgery	7.0 (5.63–8.37)			
Interventional Radiology	8.0 (7.19–8.81)			
**Type of interventional Radiology**		0.378		
RFA	8.0 (6.07–9.94)			
TACE	7.0 (6.10–7.90)			
PEI	14.0 (6.27–21.74)			
**CHILD Score (At time of bone metastasis)**		***0.000***	0.369	1.138
A	9.0 (6.24–11.76)			
B	8.0 (6.94–9.07)			
C	6.0 (4.96–7.04)			
**AFP (median value atdiagnosis)**		0.297		
<43 ng\ml	9.0 (6.78–11.22)			
>43 ng/ml	7.0 (6.37–7.63)			
**Visceral Metastasis**		0.073		
Yes	7.0 (6.24–7.76)			
No	12.0 (9.53–14.47)			
**ECOG PS (at time of Bone Metastasis)**		***0.014***	0.297	1.133
**0**	12.0 (9.24–14.76)			
**1**	8.0 (7.06–8.94)			
**2**	7.0 (5.75–8.26)			
**3**	7.0 (3.02–10.98)			
**Bone Lesion Type**		0.895		
Osteolytic	8.0 (7.04–8.96)			
Ostroblastic	9.0 (5.88–12.12)			
Mixed	8.0 (7.00–9.00)			
**Bone metastasis - Spine**		***0.021***	0.767	1.062
No	10.0 (7.93–12.07)			
Yes	7.0 (6.27–7.74)			
**Bone Metastasis - Long Bones**		0.422		
No	8.0 (7.11–8.89)			
Yes	8.0 (5.74–10.26)			
**Bone Metastasis - Hip**		0.101		
No	7.0 (6.24–7.77)			
Yes	9.0 (6.21–11.79)			
**Bone Metastasis - Other**		***0.021***	***0.025***	***0.570***
No	7.0 (6.14–7.86)			
Yes	12.0 (8.38–15.62)			
**Biphosphonate Treatment**		0.578		
No	4.0 (1.38–6.62)			
Yes	7.0 (5.99–8.01)			
**Sorafenib Treatment**		0.650		
No	3.0 (0.00–7.83)			
Yes	6.0 (4.65–7.35)			

### Skeletal outcomes and SREs according to time of bone metastases appearance

The entire population was divided in three subpopulations (synchronous bone metastases, metachronous bone metastases and patients with only bone metastases) and each subgroups was characterised for the following parameters: clinical, pathological and bone metastases characteristics, SREs and skeletal outcomes.

The three groups were homogeneous for age, gender, visceral metastases, type, site and number of bone lesions and type and number of SRE. Interestingly, median survival after bone metastases diagnosis resulted the same (7 months) in the three groups of patients, indicative of the poor prognosis strictly related to the presence of bone disease in HCC patients.

## Discussion

Bone is an uncommon site of metastasis in HCC, with the incidence ranging from 3% to 20% [11]–[14]. Anyway bone involvement in patients with HCC is increased in the last decades probably due to the longer survival of HCC patients related to recent progresses made both in the diagnosis and treatment of the disease [14], [15]. Some retrospective studies with a limited number of patients have described the characteristics of bone metastasis from HCC [11]–[16]. To our knowledge, this study is the recent largest multicentre survey investigating the natural history of metastatic bone disease in patients with HCC. Approximately less than one third presented bone metastasis at the time of initial HCC diagnosis, whereas the others developed bone metastasis during disease progression. Interestingly, median survival after bone metastases diagnosis resulted the same in both groups (7 months). Moreover, these two populations of bone metastatic HCC patients did not show any significant difference in terms of clinical, pathological and bone metastases characteristics, SREs and skeletal outcomes. The lack of differences could be indicative of the poor prognosis associated with the presence of bone disease in HCC patients. Among all the clinical and pathological parameters predicting the appearance of metastasis, only the Child Score resulted independently correlated early bone progression. This is the first report that indicates the Child Score not only as a predictor of overall survival, but also of a greater tendency to bone metastatization and biological osteotropism of HCC. The most common site (spine) and type (osteolytic) of bone metastasis are consistent with previous smaller reports and were confirmed in this study [14], [16]. Moreover, we found in the multivariate analysis that the localization of bone metastasis to spine is correlated with a shorter survival after development of first SRE and, in addition, a shorter overall survival from HCC diagnosis. This is quite different from the analysis of other bone metastasic hystotypes and from the previous reports in HCC [17]. Thus, we have found that the site of metastasis is correlated with survival, whereas the number of bone lesions do not.

Prospective data on the efficacy of bisphosphonates in bone metastatic HCC are lacking in literature [18]. This study revealed also that the bisphosphonate treatment impact on survival from the diagnosis of bone metastasis but, surprisingly, not on time to first SRE. It is possible that this result may be influeced by a selection bias exposed in the limitations of this study. Limitations of this study include its retrospective design and inclusion of an unselected heterogeneous cohort of patients with all types of aetiologic variants of HCC, liver function variants (Child score) as well as only approximately more than half of patients have been treated with Sorafenib. However, the types of patients included in this study represent the typical scenario of a real clinical practice. Another limitation is the heterogeneity of standardized methods used for detecting bone metastases, with each methodology having its own limit of detection. In summary, the results presented in this multicenter survey represent a significant improvement in the understanding the natural history of skeletal disease in HCC patients. In particular, we showed that the presence of bone metastases should always be considered in patients with a worst Child Score, even in the absence of clear symptoms, due to associated greater biological osteotropism. Second, the site of bone metastasis but not the number of the lesions is an important prognostic factor of survival from first SRE and, surprisingly, of overall survival. With impact on clinical practice, our results showed also that the use of bisphosphonates has an impact on survival from the diagnosis of bone metastasis in this population and, even if the study was unpowered to demonstrate that, bisphosphonates therapy should be considered.

The major limitation of this study is the absence of the control group. In fact, this study was designed as a retrospective observational study, aimed to describe only the natural history of HCC patients with bone metastases.

Finally, we found a significantly longer median survival after bone metastases diagnosis (7 months) compared to previous reports [14], [17]. This is extremely important since longer survival means augmented risk of SRE e subsequent worsening in quality of life (QOL).
